# Physician Network Breadth in Medicare Advantage Plans Offering Part B Premium Givebacks

**DOI:** 10.1001/jamanetworkopen.2025.55028

**Published:** 2026-01-23

**Authors:** Matthew Lavallee, Andrew Anderson, Laura J. Samuel, Kali S. Thomas, Mark K. Meiselbach

**Affiliations:** 1 Department of Health Policy and Management, Johns Hopkins Bloomberg School of Public Health, Baltimore, Maryland; 2 Johns Hopkins School of Nursing, Baltimore, Maryland

## Abstract

This cross-sectional study examines whether physician network breadth differs between Medicare Advantage giveback plans and nongiveback plans.

## Introduction

In 2023, more than one-third of Medicare enrollees reported forgoing care due to cost concerns,^1^ and for approximately 20% of enrollees, Part B premiums consumed more than 8% of their annual income.^2^ Unlike copayments or deductibles, the Part B premium is withdrawn from Social Security payments regardless of service use.

Medicare Advantage (MA), which now enrolls most Medicare beneficiaries, has increasingly used rebate dollars from the Centers for Medicare & Medicaid Services (CMS) to offer Part B premium givebacks that lower the Part B premiums for enrollees.^3^ Prior research suggests that these giveback plans enroll healthier beneficiaries and impose higher cost-sharing.^3^ However, less is known about how these plans differ in other dimensions of benefit design. In this study, we examined whether physician network breadth differs between giveback plans and nongiveback plans.

## Methods

The Johns Hopkins University Institutional Review Board exempted this cross-sectional study from review and informed consent owing to the use of publicly available data. We followed the STROBE reporting guideline. Data were collected from April 1, 2025, to Novembe 30, 2025.

We constructed county-level physician networks by specialty and plan. We first identified practicing physicians likely to participate in MA using the 2021 OneKey reference file (IQVIA).^4^ We then used a 2024 provider-network file (Ideon)^5^ to connect physicians with the plans with which they contract. We included networks with quality data only (eMethods in Supplement 1). The resulting plan county-level data captures 78% of health maintenance organization and preferred provider organization enrollment (eTable in Supplement 1).

Our outcome measure was network breadth, defined as the proportion of physicians in a county contracted by a particular plan and calculated among all physicians, primary care physicians, all specialists, and each individual CMS-regulated specialty. First, we examined the association between county-level plan network breadth and the presence of a Part B premium giveback. Second, we assessed the dose-response association between the giveback amount (above or below the median) with network breadth relative to nongiveback plans. All analyses were weighted by county-level plan enrollment, and regression analyses controlled for county and plan type fixed effects. Statistical analyses were performed in R, version 4.4.1 (R Project for Statistical Computing). Two-sided *P* < .05 indicated statistical significance.

## Results

The Table presents enrollment-weighted means of network breadth for giveback and nongiveback plans. Giveback plans included a share of total physicians of −3.94 (95% CI, −4.59 to −3.30) percentage points (pp) compared with nongiveback plans (an approximately 7% lower share), adjusting for plan type and county. In 23 of 25 individual specialties, giveback plans had significantly narrower networks (range, 1.75-5.21 pp; *P* < .05 across all comparisons); psychiatry was the only specialty where networks were significantly broader (2.24 pp [95% CI, 0.88-3.59]; *P* = .001) in giveback plans.

**Table.** Medicare Advantage Physician Network Breadth by Presence of a Part B Premium Giveback Benefit^a^

Physician type	Network breadth, mean (SE)		Difference (95% CI)	Adjusted difference (95% CI)^d^
	Part B giveback benefit (n = 191 361)^b^	No Part B giveback benefit (n = 562 035)^c^		
All physicians	52.82 (0.59)	55.99 (0.43)	−3.17 (−3.64 to −2.71)	−3.94 (−4.59 to −3.30)
Primary care	50.64 (0.61)	54.81 (0.43)	−4.17 (−4.64 to −3.69)	−4.43 (−5.10 to −3.77)
All specialists	58.42 (0.68)	59.62 (0.50)	−1.20 (−1.77 to −0.63)	−3.04 (−3.74 to −2.34)
CMS-regulated specialty				
Allergy and Immunology	59.20 (1.58)	60.51 (1.00)	−1.32 (−2.75 to 0.11)	−2.98 (−5.21 to −0.74)
Cardiology	69.28 (0.93)	68.58 (0.69)	0.71 (−0.16 to 1.57)	−2.66 (−3.64 to −1.68)
Cardiothoracic surgery	58.94 (1.28)	58.63 (0.92)	0.32 (−0.97 to 1.60)	−2.39 (−4.01 to −0.76)
Dermatology	61.59 (1.23)	65.38 (0.79)	−3.79 (−4.86 to −2.72)	−5.10 (−6.57 to −3.62)
Endocrinology	60.87 (1.27)	62.67 (0.87)	−1.80 (−3.01 to −0.59)	−4.69 (−5.94 to −3.44)
ENT or otolaryngology	61.01 (1.16)	62.30 (0.77)	−1.29 (−2.29 to −0.28)	−4.02 (−5.30 to −2.74)
Gastroenterology	67.80 (1.04)	67.94 (0.75)	−0.14 (−1.12 to 0.84)	−3.51 (−4.47 to −2.55)
General surgery	59.06 (0.77)	60.16 (0.56)	−1.10 (−1.76 to −0.44)	−3.13 (−3.88 to −2.38)
Gynecology or OB-GYN	56.50 (0.89)	60.38 (0.59)	−3.87 (−4.59 to −3.15)	−5.21 (−6.15 to −4.28)
Infectious diseases	55.34 (1.34)	57.82 (0.89)	−2.49 (−3.72 to −1.26)	−3.74 (−5.24 to −2.23)
Nephrology	68.94 (1.07)	68.07 (0.76)	0.86 (−0.12 to 1.85)	−1.27 (−2.64 to 0.10)
Neurology	51.28 (1.09)	54.59 (0.73)	−3.31 (−4.27 to −2.35)	−3.44 (−4.49 to −2.40)
Neurosurgery	58.08 (1.37)	58.54 (0.97)	−0.46 (−1.85 to 0.93)	−1.99 (−3.90 to −0.08)
Oncology (medical)	65.91 (1.08)	65.16 (0.76)	0.76 (−0.23 to 1.74)	−2.42 (−3.41 to −1.43)
Oncology (radiation)	61.69 (1.30)	61.77 (0.92)	−0.08 (−1.31 to 1.15)	−3.31 (−4.71 to −1.91)
Ophthalmology	63.64 (1.11)	67.28 (0.70)	−3.65 (−4.56 to −2.73)	−5.07 (−6.97 to −3.17)
Orthopedic surgery	62.85 (0.93)	63.95 (0.65)	−1.11 (−1.91 to −0.31)	−3.60 (−4.71 to −2.49)
Physiatry	50.40 (1.20)	54.80 (0.79)	−4.40 (−5.46 to −3.33)	−5.03 (−6.05 to −4.02)
Plastic surgery	44.34 (1.53)	47.01 (1.01)	−2.67 (−4.11 to −1.22)	−4.36 (−5.98 to −2.74)
Psychiatry	34.96 (1.01)	31.31 (0.72)	3.65 (2.76 to 4.54)	2.24 (0.88 to 3.59)
Pulmonology	59.08 (1.08)	60.53 (0.75)	−1.44 (−2.42 to −0.46)	−2.91 (−4.09 to −1.74)
Rheumatology	61.21 (1.37)	62.98 (0.91)	−1.77 (−3.03 to −0.50)	−4.96 (−6.57 to −3.36)
Urology	64.67 (1.12)	65.20 (0.75)	−0.53 (−1.50 to 0.45)	−3.39 (−4.37 to −2.42)
Vascular surgery	64.87 (1.27)	64.13 (0.89)	0.74 (−0.50 to 1.98)	−1.75 (−3.15 to −0.35)

Abbreviations: CMS, Centers for Medicare & Medicaid Services; ENT, ear, nose, and throat; OB-GYN, obstetrics and gynecology.

^a^ Network breadth is measured as the proportion of physicians in a county contracted by a given plan. All values are enrollment weighted, and SEs are clustered at the county level. Data are from the 2021 OneKey reference file (IQVIA) and the 2024 provider-network file (Ideon).

^b^ Among 2 million enrollees.

^c^ Among 14.2 million enrollees.

^d^ Adjusted for county fixed effects and plan type (health maintenance organization vs preferred provider organization).

The Figure shows the association between the giveback amount and network breadth for all physicians, primary care physicians, and specialists. Both below- and above-median giveback plans were associated with lower network breadth compared with plans without givebacks. These differences were greater among plans with above-median giveback amounts than in plans with below-median giveback amounts.

**Figure.**
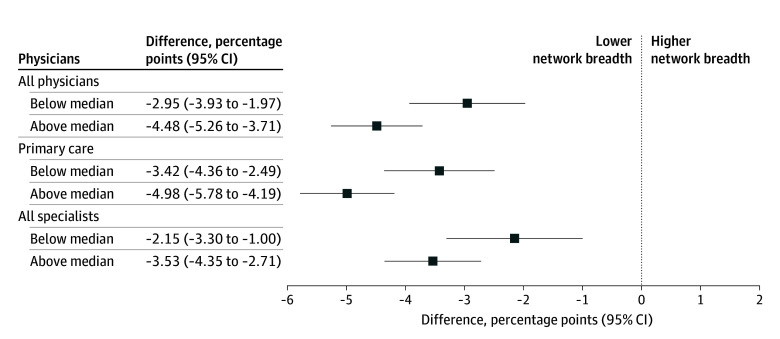
Association Between Medicare Advantage Part B Giveback Amount and Specialist Network Breadth by Giveback Amount Network breadth is the proportion of physicians in a county contracted by a given plan. Estimated differences in network breadth were adjusted for county-level fixed effects and plan type (health maintenance organization vs preferred provider network) and weighed by county-level enrollment. SEs are clustered at the county level. Below median giveback amount = $1 to $74; above median giveback amount = $75 to $165. Data are from the 2021 OneKey reference file (IQVIA)^4^ and the 2024 provider-network file (Ideon).^5^

## Discussion

MA plans offering Part B premium givebacks had narrower physician networks than those without this benefit, consistent across most individual specialties. Among giveback plans, those with more generous giveback amounts had narrower networks than those offering smaller amounts. These findings suggest that patients choosing plans may face trade-offs between receiving Part B premium rebates and having access to broader physician networks, an aspect of plan design that can be difficult for beneficiaries to evaluate. Prior studies have also found that Part B premium giveback plans have higher cost-sharing than nongiveback plans.^3^

Our study is limited in that it only examines 1 dimension of clinician access, omitting access to nonphysician clinicians and health care facilities. As policymakers consider how to regulate MA, they will need to ensure consumers are well-informed of trade-offs faced between supplemental cash benefits and plan features that may limit access to care.

## Notes

Supplement 1. **eMethods.** Provider and County-Plan Sample Inclusion **eTable.** County-Plan Selection Criteria

Supplement 2.
Data Sharing Statement
