# Supplementary material for: Physician Network Breadth in Medicare Advantage Plans Offering Part B Premium Givebacks
Source: JAMA Netw Open. 2026 Jan 23;9(1):e2555028. doi: 10.1001/jamanetworkopen.2025.55028 (PMC12831149; doi:10.1001/jamanetworkopen.2025.55028)
Supplement: Supplement 1. — eMethods. Provider and County-Plan Sample Inclusion eTable. County-Plan Selection Criteria [file jamanetwopen-e2555028-s001.pdf]

Supplementary Online Content

Lavallee M, Anderson A, Samuel LJ, Thomas KS, Meiselbach MK. Physician network breadth in Medicare Advantage plans offering Part B premium givebacks. *JAMA Netw Open.* 2026;9(1):e2555028. doi:10.1001/jamanetworkopen.2025.55028

eMethods. Provider and County-Plan Sample Inclusion

eTable. County-Plan Selection Criteria

This supplementary material has been provided by the authors to give readers additional information about their work.

© 2026 Lavallee M et al. *JAMA Network Open*.

eMethods. Provider and County-Plan Sample Inclusion

Provider Sample Inclusion

To construct measures of network breadth, we first identify physicians likely to participate in Medicare Advantage. We use IQVIA's 2021 OneKey reference file and apply the following exclusion criteria: physicians must (1) hold an MD or DO degree, (2) have a valid National Provider Identifier (NPI), (3) be born after 1940, (4) report a practice setting within scope (e.g., hospital, insurance, outpatient, residential), (5) not serve in the armed forces, (6) not be a student healthcare worker, and (7) report accepting Medicare. After applying all criteria, we retain 71% of MD/DO providers in OneKey, with Medicare acceptance being the most restrictive filter. This results in 757,027 unique physicians.

We then link physicians to CMS-regulated specialties using NPI-taxonomy code combinations from the National Plan and Provider Enumeration System and a CMS-provided taxonomy-to-specialty crosswalk. Of the 757,027 physicians, 537,741 (71%) match to at least one CMS-recognized specialty.

Finally, we link physicians to Medicare Advantage networks using the 2024 Ideon provider-network file. All physicians from our set of 537,741 specialists link to at least one network contract, resulting in a final sample of 537,471 physicians for measuring network breadth.

County-Plan Sample Inclusion

To ensure that we measure networks only for plans actively serving enrollees, we construct a county-level plan sample using CMS's 2024 Q3 Plan Benefit Package (PBP) data. We include county-plan combinations that meet eight criteria: (1) the plan has nonzero national enrollment; (2) the plan is classified as an HMO or PPO; (3) the county is located in one of the 50 U.S. states (excluding Alaska); (4) the county-plan has more than 11 enrollees; (5) the county contains at least one physician in the OneKey database; (6) the plan links to the Ideon provider-network file; (7) the plan contracts with at least one physician in our final sample of 537,471 providers; and (8) the plan's network breadth in their service area exceeds 2%. After applying these criteria, we retain 78% of national HMO/PPO enrollment. A summary of sample construction, including enrollment in giveback plans, is shown in eTable 1 and aligns with previous work.

© 2026 Lavallee M et al. *JAMA Network Open*.

eTable. County-Plan Selection Criteria

Criteria	Total Enrollment	Share of HMO/PPO Enrollment
>0 National Enrollment	20,843,347	1.00
Eligible State	20,623,170	0.99
>11 County Enrollment	20,843,347	1.00
Has OneKey	20,810,474	1.00
Links to Ideon	20,623,170	0.99
Contracts with Ideon Physicians	20,257,147	0.98
>2% Specialist Service Area Breadth	16,228,204	0.79
All Criteria	16,198,794	0.78

© 2026 Lavallee M et al. *JAMA Network Open*.
