# Supplementary material for: Physician Network Breadth in Medicare Advantage Plans Offering Part B Premium Givebacks
Source: JAMA Netw Open. 2026 Jan 23;9(1):e2555028. doi: 10.1001/jamanetworkopen.2025.55028 (PMC12831149; doi:10.1001/jamanetworkopen.2025.55028)
Supplement: Supplement 2. — Data Sharing Statement [file jamanetwopen-e2555028-s002.pdf]

Data Sharing Statement

Lavallee. Physician Network Breadth in Medicare Advantage Plans Offering Part B Premium Givebacks. *JAMA Netw Open*. Published January 23, 2026.
doi:10.1001/jamanetworkopen.2025.55028

Data
Data available: No
